# Generative AI‐Driven Accelerated Discovery of Passivation Molecules for Perovskite Solar Cells

**DOI:** 10.1002/advs.202523042

**Published:** 2026-04-02

**Authors:** Adroit T. N. Fajar, Guillaume Lambard, Jessie Manopo, Ruili Guo, Kevin Septioga, Rizfi F. Pari, Toshinori Matsushima, Zhanglin Guo

**Affiliations:** ^1^ International Institute for Carbon‐Neutral Energy Research (WPI‐I^2^CNER) Kyushu University Fukuoka Japan; ^2^ Center for Energy Systems Design (CESD) International Institute for Carbon‐Neutral Energy Research (WPI‐I^2^CNER) Kyushu University Fukuoka Japan; ^3^ Data‐Driven Materials Design Group Center for Basic Research on Materials National Institute for Materials Science Tsukuba Japan; ^4^ Department of Applied Chemistry Graduate School of Engineering Kyushu University Fukuoka Japan; ^5^ Department of Automotive Science Graduates School of Integrated Frontier Sciences Kyushu University Fukuoka Japan

**Keywords:** generative AI, inverse design, perovskite solar cells, passivation molecules, SMILES

## Abstract

Molecular passivation is an effective strategy to suppress interfacial defects in perovskite solar cells (PSCs), yet the discovery of new passivation molecules remains limited by empirical design and narrow chemical libraries. Here, for the first time, we present an AI‐driven framework integrating discriminative and generative language models to accelerate the discovery of effective passivators. A SMILES‐X classifier trained on literature data achieved high predictive performance (F1 = 0.80, ROC–AUC = 0.88), while a GPT‐2‐based generative model iteratively produced over 100 000 novel molecules, more than 80% of which were predicted to be effective. Multi‐criteria filtering reduced this pool to ∼8000 high‐quality candidates, from which clustering analysis identified ten diverse representatives. Three molecules, including a surrogate analog, were prioritized for experimental testing, and all exhibited a clear passivation effect. In particular, 4‐maleimidobutyric acid increased the average open‐circuit voltage from 1.08 to 1.12 V and improved the average power conversion efficiency from 19.3% to 22.2%, while markedly reducing hysteresis. This study demonstrates that generative AI can autonomously propose synthetically accessible, functionally effective molecules for PSC passivation, offering a powerful paradigm for accelerated materials discovery beyond conventional chemical space exploration.

## Graphical abstract

A generative artificial intelligence (AI) framework combining a discriminative machine learning model (SMILES‐X) and a generative language model (GPT‐2) autonomously discovers new molecular passivators for perovskite solar cells (PSCs). Through an iterative design loop, over 100 000 candidates are generated and screened, and randomly selected molecules are experimentally validated, demonstrating reliable AI‐driven materials discovery for PSCs.

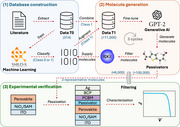

## 1 Introduction

Halide perovskite solar cells (PSCs) have emerged as one of the most promising next‐generation photovoltaic technologies owing to their exceptional optoelectronic properties, facile solution processability, and tunable bandgaps. Since their initial demonstration with a modest power conversion efficiency (PCE) of 3.8%, the efficiency of single‐junction PSCs has rapidly advanced to 27.0%, while perovskite‐based tandem devices are now approaching 35% [1, 2]. Despite this remarkable progress, further improvements in efficiency and operational lifetime are hindered by interfacial and surface defects that promote nonradiative recombination and ion migration.

Surface defect passivation using small organic molecules has proven to be one of the most effective strategies to mitigate these issues. A representative example is phenethylammonium iodide, which binds to undercoordinated Pb^2+^ or halide vacancies and can form quasi‐2D capping layers, thereby improving efficiency [3, 4]. Numerous other functional molecules bearing amine, thiol, carbonyl, or carboxyl groups have also been reported to interact with the perovskite lattice and suppress defects [5]. However, the discovery of new passivation molecules has relied heavily on empirical trial‐and‐error chemistry, which is time‐consuming, costly, and often lacks clear design principles. A more efficient and systematic approach is therefore urgently needed.

Machine learning (ML) has been used as a powerful tool to accelerate molecule discovery by learning structure–property relationships from large datasets. Early examples include supervised models trained on 21 organic–halide capping layers, which revealed that a low hydrogen‐bond donor count and small polar surface area could extend perovskite film lifetime fourfold [6]. Subsequent studies employing random forest regressors and DFT‐derived binding energy filters identified NH_3_^+^‐terminated cations such as 2‐phenylpropane‐1‐aminium iodide, boosting device efficiency to 24.5% [7]. More advanced physics‐informed neural networks trained on full‐DFT datasets screened five million pseudo‐halide anions and discovered sodium thioglycolate, achieving 24.6% PCE with 96% retention after 900 h [8]. Ensemble learning and complexity‐weighted descriptors, respectively, guided the selection of sulfonated and carboxyphthalide‐based candidates that enhanced device efficiency [9, 10]. Despite these advances, conventional ML approaches remain inherently limited by their reliance on predefined chemical libraries and labeled datasets, restricting exploration to known molecular spaces rather than enabling the generation of entirely new chemical structures with unconventional functionalities.

Generative artificial intelligence (AI) offers a transformative paradigm shift, from screening known compounds to inverse molecular design. By leveraging sequence‐ or graph‐based architectures, generative AI can navigate vast chemical spaces, estimated to contain more than 10^60^ synthetically accessible small molecules [11], and propose novel compounds beyond existing databases. Recent reinforcement learning frameworks, such as SyntheMol, have successfully generated and experimentally validated new antibiotic candidates [12]. Similarly, in materials science, diffusion‐based models like MatterGen have demonstrated more than a twofold increase in discovering stable crystal structures compared with traditional generators [13]. However, applications of generative AI to the discovery of molecular passivators for perovskite photovoltaics remain largely unexplored [14, 15]. Establishing such AI‐driven pipelines could enable the design of previously unknown molecular scaffolds with superior binding affinities, defect‐healing capabilities, and photothermal robustness, which are key requirements for achieving stable, high‐efficiency PSCs.

In this study, we present a language model (LM)‐based generative framework for the discovery of molecular passivators for perovskite solar cells. Specifically, for the first time, we integrate fine‐tuned large language models (LLMs, e.g., GPT‐2 and LLaMA‐2) with an LM‐based molecular characterization tool (SMILES‐X) and employ an iterative generation–screening pipeline to produce over 100 000 novel candidate molecules. The generated molecules are filtered using seven physicochemical criteria and grouped into ten fingerprint‐based clusters, from which one representative molecule is sampled per cluster to ensure chemical diversity. After expert evaluation, three representative molecules were experimentally tested in PSC devices. All three AI‐generated molecules effectively improved the open‐circuit voltage (*V*_OC_) of inverted PSCs, demonstrating their ability to passivate perovskite surface defects. Notably, 4‐maleimidobutyric acid (MBA) not only enhanced *V*_OC_ but also optimized interfacial energy alignment by eliminating electron‐transport barriers, thereby minimizing energy losses and reducing *J*–*V* hysteresis. These results demonstrate the potential and reliability of AI‐driven molecular generation for accelerated materials discovery in perovskite photovoltaics, offering a powerful new pathway to explore the vast, uncharted chemical space for next‐generation solar energy materials.

## 2 Results and Discussion

**SCHEME 1:**
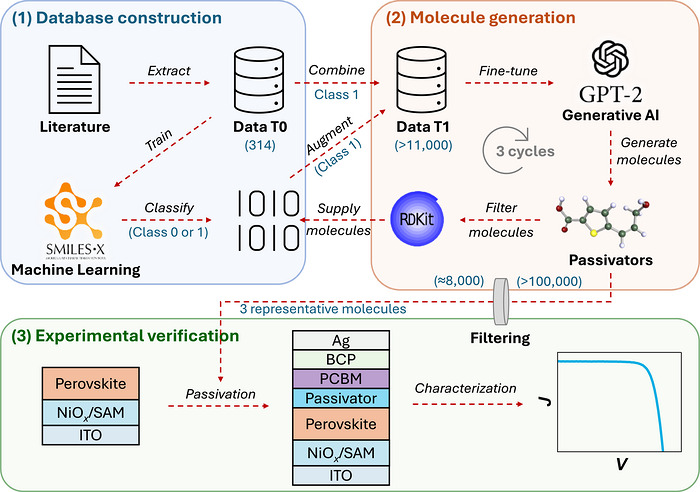
Workflow of AI‐driven novel passivation molecules discovery and verification.

The workflow for discovering novel passivation molecules using the generative AI framework is illustrated in Scheme 1. It begins with the construction of an initial database (Data T0, 314 molecules) consisting of experimentally reported molecules collected from the literature, each labeled according to its relative (normalized) improvement in power conversion efficiency (Δ*PCE*_norm_, see Equation S1). This dataset was used to train a discriminative model, SMILES‐X [16], which directly interprets molecular SMILES representations to classify molecules as either effective (class 1) or ineffective (class 0) passivators. The trained model was then employed to predict new candidates with high structural similarity to the top‐performing molecules, thereby augmenting the original training data. In the second stage, a generative language model (GPT‐2) was fine‐tuned on the subset of effective molecules from the augmented dataset (Data T1) to autonomously generate new molecular structures with potential passivation capability. Through three iterative training and inference cycles, the model produced over 100 000 molecules, of which more than 80% were predicted to belong to class 1. The generated molecules were subsequently filtered based on physicochemical and structural criteria, yielding a refined set of ∼8000 promising candidates. These were further grouped into ten clusters according to structural similarity. Finally, one representative molecule was selected from each cluster, and based on expert evaluation, three were chosen for experimental testing. The following sections describe the detailed processes of this study and the results obtained.

### 2.1 Existing Passivation Molecules

**FIGURE 1:**
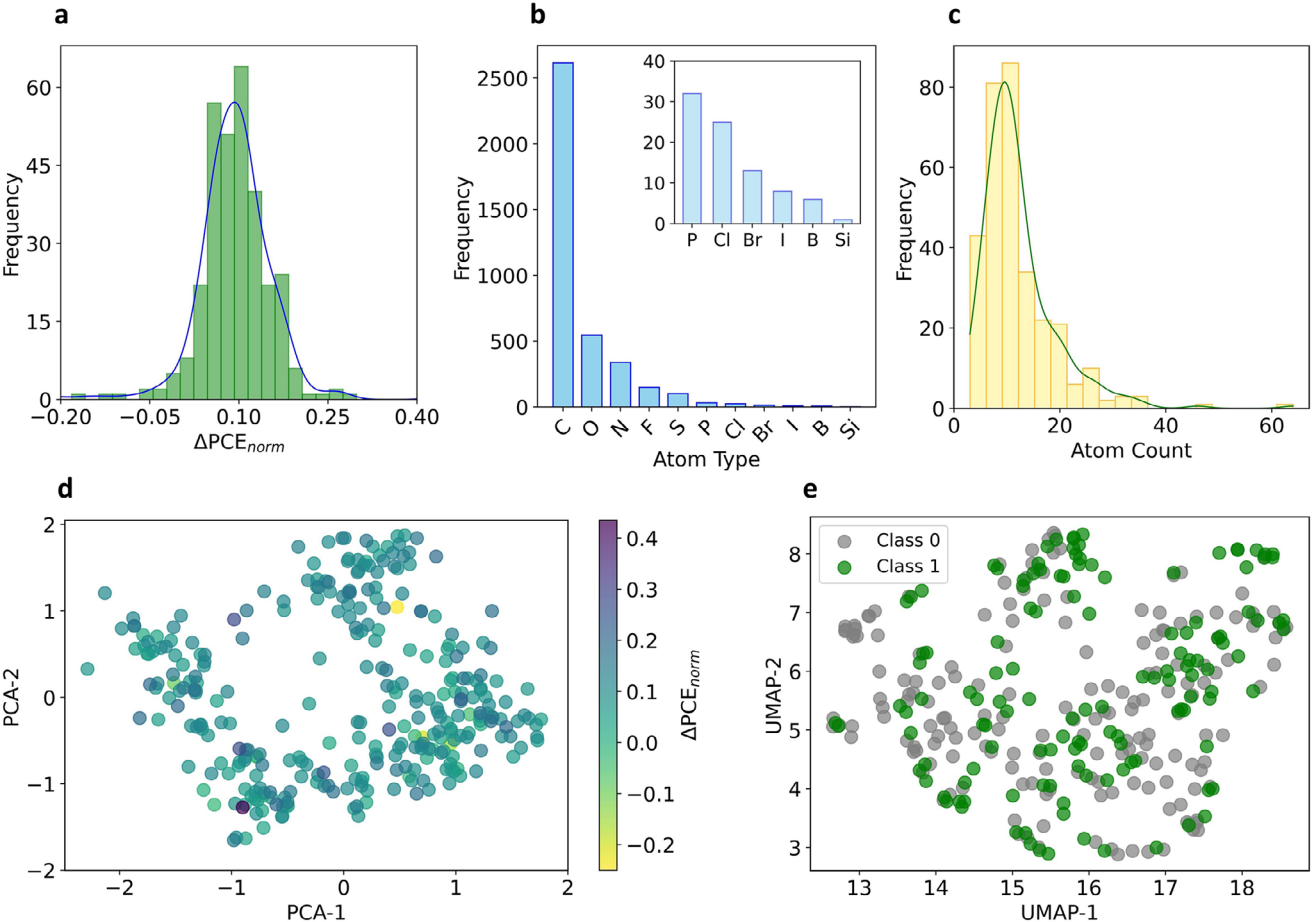
Characteristics of the existing passivation molecules. Distribution of (a) passivation effects (Δ*PCE*_norm_ values), (b) atom types, and (c) atom counts in the dataset (Data T0). Chemical space visualization of the dataset using (d) PCA labeled with Δ*PCE*_norm_ values, and (e) UMAP labeled with binary classification results.

Understanding existing data is a crucial first step in building an AI system for any specific application. In the field of PSCs, hundreds of passivation molecules have been reported over the past decade (see Data T0). Data T0 was constructed by systematically mining previously reported passivation molecules from curated review articles and related primary studies, followed by manual verification of the original publications. The dataset was rigorously preprocessed to remove duplicates, convert all structures to canonical SMILES, and extract paired initial/final PCE values, yielding 314 unique labeled molecules (see Supporting Information). As shown in Figure 1a, most reported molecules result in moderate improvements in device performance (normalized PCE improvement, Δ*PCE*_norm_ ≈ 0.10). A few molecules exhibit negative effects (typically reported as controls in the original studies), while some demonstrate exceptionally positive effects. Among these molecules, electronegative atoms (i.e., O, N, F, S) are commonly utilized to induce defect passivation (Figure 1b). For example, functional groups with a higher oxygen content have been reported to exhibit stronger binding energy toward undercoordinated Pb^2+^ defects on the perovskite surface through the Lewis acid–base coordination [8]. Similarly, nitrogen‐containing functional groups can interact via hydrogen bonding with FA^+^/MA^+^ cations, contributing to defect suppression [17]. Figure 1c presents the distribution of atom counts in the dataset, reflecting molecular size. Small molecules (< 20 atoms) are most frequently used for surface passivation, whereas the largest reported molecules contain up to 60 atoms.

To further characterize the dataset, principal component analysis (PCA) and uniform manifold approximation and projection (UMAP) were applied to the Morgan fingerprints of the molecules. In these plots, PCA‐1 and PCA‐2 correspond to the first and second principal components, which capture the most significant directions of variation in the molecular feature space. They help visualize how molecules differ based on their overall structural characteristics. Similarly, UMAP‐1 and UMAP‐2 are the two main dimensions obtained from the UMAP projection, which preserves both local and nonlinear relationships between molecules, making it easier to observe potential clustering patterns in the dataset [18]. Figure 1d shows the 2D PCA projection of the molecules, labeled by their Δ*PCE*_norm_ values. This analysis provides insight into global variance relationships among molecules. However, molecular structures with low, moderate, and high passivation effects are not clearly separated in the chemical space, suggesting that the structure–property relationship governing passivation is highly complex. Figure 1e displays the 2D UMAP projection with binary classification labels, where class 0 represents ineffective passivation (Δ*PCE*_norm_ < 0.10) and class 1 represents effective passivation (Δ*PCE*_norm_ ≥ 0.10). Although UMAP better preserves local structures and non‐linear relationships than PCA, distinct class groupings are still not observed. These analyses indicate that identifying suitable molecular scaffolds or substructures for perovskite passivation is inherently challenging, even for human experts (see Figure S1), and thus strongly motivates the application of ML‐based approaches.

### 2.2 Expanding the Chemical Space

**FIGURE 2:**
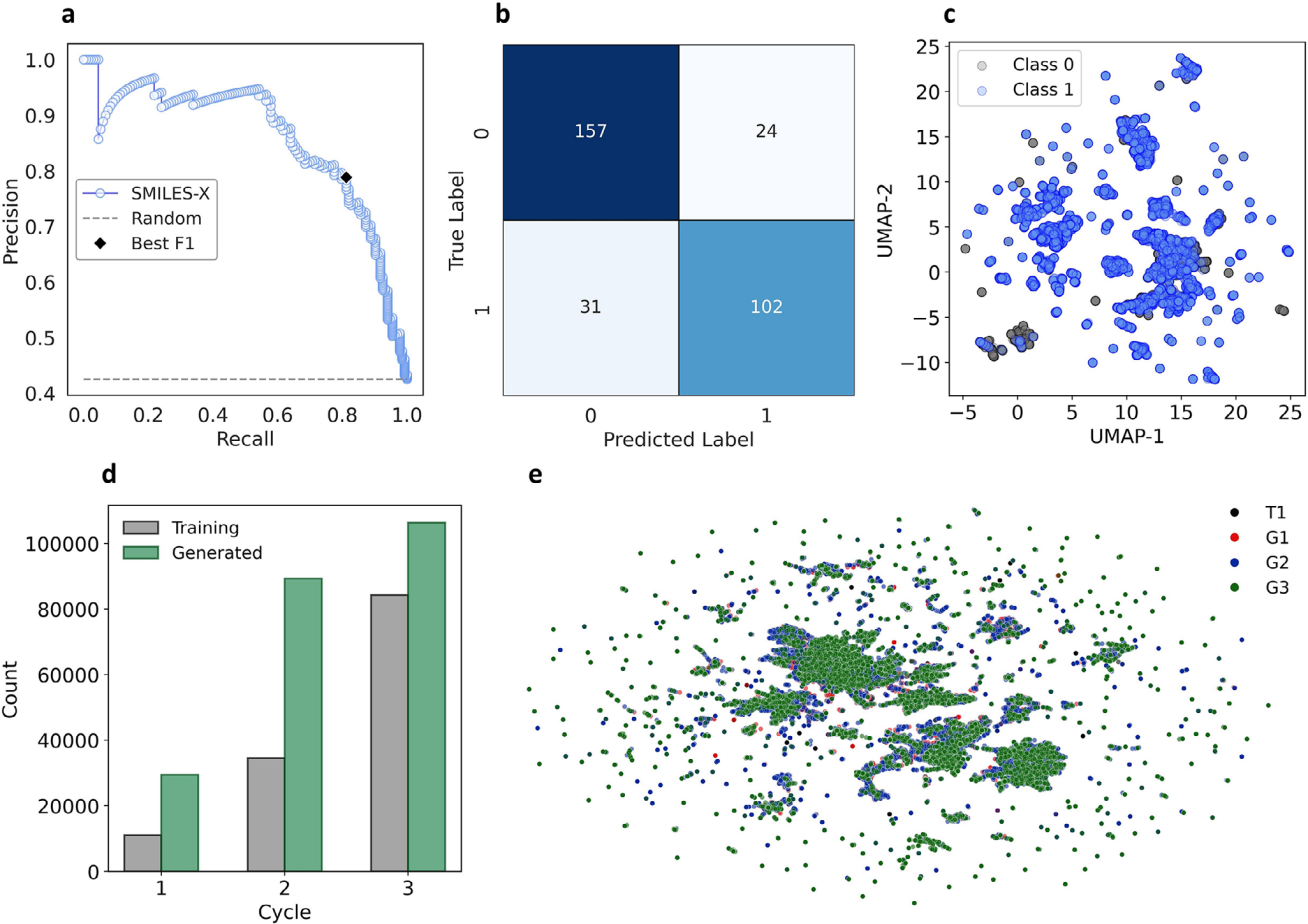
Expanding the chemical space of PSC passivation molecules using language models. (a) Precision–recall (PR) curve and (b) confusion matrix of the binary classification model prepared with SMILES‐X trained on Data T0. (c) UMAP projection of Data T‐aug labeled with binary classification results predicted by SMILES‐X. (d) Number of molecules and (e) chemical space visualization across three cycles of iterative training and generation with the fine‐tuned GPT‐2 model.

Building accurate ML prediction models for molecular structure–property relationships typically requires rigorous feature extraction by domain‐specific experts or quantum chemical calculations [19, 20], which are time‐consuming and resource‐intensive. Following the principle of natural language processing, SMILES‐X bypasses this step by directly using the SMILES string as input (optionally augmented with a few simple descriptors) and mapping it to property labels [16]. Here, a binary classification model was developed to distinguish ineffective (class 0) and effective (class 1) passivation molecules by training SMILES‐X on Data T0. To evaluate model performance, both the precision–recall (PR) curve and confusion matrix were analyzed based on the final out‐of‐sample predictions. As shown in Figure 2a, the PR curve illustrates the trade‐off between precision and recall across a range of probability thresholds (mean scores). The optimal threshold of 0.47 yielded the maximum F1 score of 0.80.

The corresponding confusion matrix (Figure 2b) provides a detailed breakdown of prediction outcomes aggregated over fivefold cross‐validation: 157 true negatives (TN), 102 true positives (TP), 31 false negatives (FN), and 24 false positives (FP). These results indicate that the model performs well in distinguishing between the two classes, with relatively low misclassification rates. Furthermore, the precision, recall, and F1‐score averaged 0.82 based on cross‐validation, confirming robust performance across folds. The area under the precision–recall curve (PRC–AUC) was 0.86, and the ROC–AUC reached 0.88, highlighting strong discriminative ability even for an imbalanced dataset. This performance is comparable to that of a binary classification model prepared using the random forest (RF) algorithm with Morgan fingerprints as input features (see Figure S2). However, unlike the RF approach, which requires explicit feature extraction, SMILES‐X is computationally efficient and can be seamlessly integrated with LM–based generative frameworks. Therefore, the developed model achieves both high predictive accuracy and computational efficiency, making it suitable for guiding AI‐driven molecular design.

Because molecular structures encoded as SMILES can be regarded as a chemical language [21], they can also be directly used to fine‐tune LLMs. However, the number of reported passivation molecules in Data T0 is relatively small and insufficient for LLM fine‐tuning. To address this, the dataset was augmented by retrieving molecules from the PubChem database with at least 80% Tanimoto similarity to the top‐performing class 1 molecules (Δ*PCE*_norm_ > 0.16), yielding 15 540 additional entries (Data T‐aug). As shown in Figure 2c, approximately 70% of these molecules were classified as class 1 by the SMILES‐X model. A new dataset combining class 1 molecules from Data T0 and Data T‐aug was then defined as Data T1 and was used to fine‐tune both GPT‐2 and LLaMA‐2 models. As proven in the following, this method largely improved the reliability and effectiveness of generative AI‐driven molecule discovery. The test loss distributions (Figure S3a,b) indicate that both models achieved low average losses (GPT‐2: 0.13; LLaMA‐2: 0.15), confirming that they successfully learned the atomic sequence patterns associated with effective passivation molecules. Despite comparable performance, LLaMA‐2 required substantially greater computational resources (five times longer for training and over 100 times slower for inference) due to its large parameter count (7 billion), which is designed for more expressive human conversation (see Methods). Consequently, the GPT‐2 model was selected for subsequent analysis.

To further expand the chemical search space of perovskite passivation molecules, the GPT‐2 model was fine‐tuned iteratively by incorporating class 1 molecules generated from each previous cycle into the training dataset (details in Methods). The iterative fine‐tuning strategy was adopted as a practical guided‐exploration approach for domain‐specific molecular discovery, where the available high‐quality training data are limited. As shown in Figure 2d, one‐shot generation (cycle 1) yields only ∼30 000 chemically valid, unique, and novel (CUN) molecules, which is insufficient to reach the targeted chemical space size. Iterative generation progressively enriches the training set with model‐prioritized candidates, allowing the number of CUN molecules to exceed 100 000 by the third cycle. The generative model expanded the dataset by approximately tenfold—from about 10 000 molecules in Data T1 to over 100 000 in Data G‐all. More than 80% (87 750) of the generated molecules were predicted by the SMILES‐X classifier to have a high model‐predicted likelihood of belonging to the effective passivation class (Data G‐class1), reflecting enrichment toward chemically promising candidates. A concise sensitivity analysis for the classifier threshold is provided in Table S1. These predictions serve as a probabilistic prioritization signal to guide exploration of the chemical space, rather than as ground‐truth labels. Consequently, further screening based on physicochemical criteria and experimental validation serves as the final validation steps. Although additional iterations could have been performed, the process was terminated after the third cycle to maintain a manageable dataset size (∼100 000 molecules) and prevent model collapse, a phenomenon observed in our previous work on ionic liquid generation and in other general‐purpose generative models [22, 23]. The atom‐type distribution for the cumulative generated molecules (Data G‐all) is shown in Figure S3c, while Figure 2e illustrates the progressive expansion of the explored chemical space across three iterative cycles relative to the original training data. To quantitatively assess structural novelty, we computed the nearest‐neighbor Tanimoto similarity between the cumulative generated library and the initial GPT‐2 fine‐tuning dataset (Figure S4). The resulting distribution indicates substantial chemical diversification beyond direct scaffold replication, with the majority of generated molecules exhibiting moderate similarity to the training set rather than near‐identical structures. Overall, this iterative fine‐tuning strategy effectively broadened the accessible molecular space for perovskite passivation, enabling exploration of previously uncharted chemical regions.

### 2.3 From AI Design to Laboratory

**FIGURE 3:**
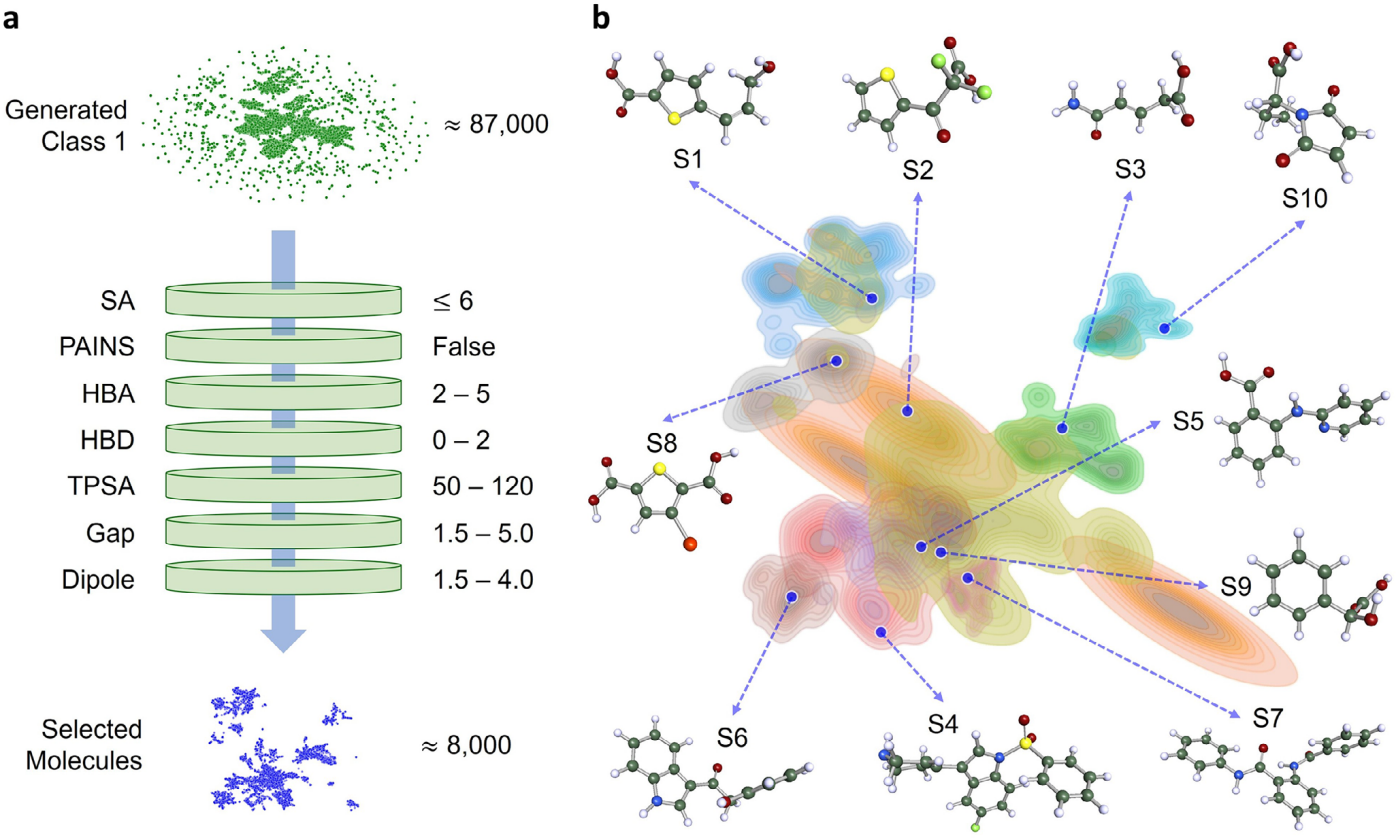
Filtering the generated molecules for laboratory assessment. (a) Schematic illustration of the filtering process based on seven physicochemical criteria. (b) Visualization of the ten agglomerative clusters of the filtered molecules, with one representative molecule randomly selected from each cluster.

With numerous perovskite passivation molecule candidates available, the next step was to select a manageable subset for laboratory analysis. To narrow down the pool, seven filtering criteria were applied. Molecules with synthetic accessibility (SA) ≤ 6 were retained to ensure practical synthesizability, while substructures known as Pan‐Assay Interference Compounds (PAINS), which often yield false‐positive results due to nonspecific reactivity or signal artifacts, were excluded. Hydrogen‐bond donor (HBD) and hydrogen‐bond acceptor (HBA) counts were restricted to 0–2 and 2–5, respectively, to balance interfacial interaction strength and molecular stability. Molecules with a topological polar surface area (TPSA) between 50 and 120 Å^2^ were selected to maintain moderate polarity suitable for thin‐film compatibility. Additionally, molecules with energy gaps between 1.5 and 5.0 eV and dipole moments between 1.5 and 4.0 D were prioritized to favor electronic stability and directional surface interactions. As illustrated in Figure 3a, this multi‐criteria filtering retained fewer than 10% of the most desirable candidates while preserving chemical diversity, yielding approximately 8000 high‐quality molecules suitable for PSC passivation. Although any molecule from this filtered set could potentially exhibit passivation activity, the molecules were further grouped into ten clusters using an agglomerative clustering algorithm to maximize structural diversity and minimize redundancy. One representative molecule was then randomly selected from each cluster for further analysis (Figure 3b), with details summarized in Table S2.

Translating AI‐generated molecules into experimentally accessible compounds remains a key bottleneck in modern molecular discovery. Because novelty is an inherent goal of generative modeling, most generated molecules are new (i.e., absent from the training data) and their synthetic feasibility is often uncertain, although SA scores offer preliminary guidance. Importantly, since the training dataset focuses on domain‐relevant molecules rather than the full chemical universe, generated candidates can occasionally coincide with existing compounds known from other research areas. This phenomenon, often termed molecular “re‐discovery,” has been widely discussed in drug discovery [24, 25]. In our randomly selected ten candidates, two known compounds: DL‐mandelic acid (S9), commonly used in antimicrobial and cosmetic formulations [26, 27], and 4‐maleimidobutyric acid (S10), a reagent for drug and protein conjugation [28], were identified. These two molecules were commercially available and thus directly selected for experimental verification. The third selected molecule, S3, appeared to be novel and required custom synthesis. To enable quick validation, a structural similarity search was conducted, identifying maleic acid monoamide (Tanimoto similarity = 85%) as a commercially available analog, which was adopted as a surrogate for S3. Details including the molecular structure of all three molecules selected for laboratory assessment are provided in Table S3.

### 2.4 Passivation Effects on PSCs

To experimentally verify the effect of the selected three representative molecules, PSCs were fabricated with an inverted configuration (Scheme 1). This structure was chosen because it offers facile fabrication and is compatible with tandem solar cell architecture, which are key for achieving higher performance. Previous studies have shown that defects, particularly those located at the perovskite upper surface and the perovskite/electron transport layer (ETL) interface [29, 30], remain the primary factors limiting the efficiency of inverted PSCs. Therefore, demonstrating the effectiveness of the selected molecules in real devices provides valuable insight for developing highly efficient PSCs. Note that experimental validation presented here should be regarded as a proof‐of‐concept demonstration of AI‐generated passivators, rather than implying broad effectiveness across all generated molecules.

As detailed in the Experimental Studies section, a 1 mg/mL passivation solution (a typical concentration for surface treatment) was prepared in 2‐propanol (IPA) and dynamically spin‐coated on the perovskite surface, followed by annealing at 100°C for 5 min. The representative current–voltage (*J*–*V*) curves of the PSCs are shown in Figure S5a. Notably, all three molecules improved the open‐circuit voltage (*V*_OC_), indicating defect passivation with suppressed nonradiative recombination at the perovskite surface. Among them, 4‐maleimidobutyric acid (MBA) yielded the most significant improvement, exhibiting a pronounced reduction in hysteresis (efficiency discrepancy between *J*–*V* curves obtained under forward scan and reverse scan) compared with both the other passivators and the control device.

**FIGURE 4:**
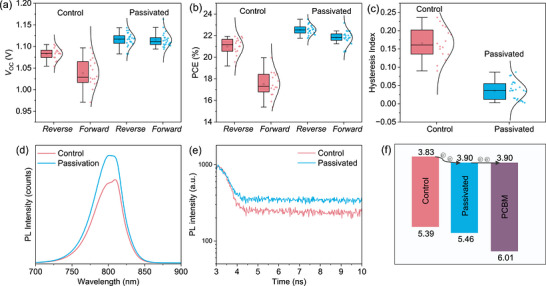
Passivation effects of 4‐maleimidobutyric acid on PSCs. (a) Open‐circuit voltage (*V*_OC_), (b) power conversion efficiency (PCE), and (c) *J*–*V* hysteresis index of PSCs with and without MBA passivation. (d, e) Steady‐state and time‐resolved photoluminescence (PL and TRPL) spectra of perovskite films with and without passivation. (f) Energy‐level diagrams of the perovskite with and without passivation and their alignment with the PCBM electron transport layer.

The optimal concentration of MBA was next examined. As shown in Figure S5b, the devices passivated with 1 mg/mL MBA achieved the highest *V*_OC_ and PCE, whereas higher concentrations slightly decreased performance, confirming 1 mg/mL as the optimal condition. These results suggest that further optimization of deposition parameters could allow other AI‐designed passivation molecules to deliver even better performance. To assess reproducibility, additional batches of devices were fabricated under this optimized condition. In the champion device, PCEs of 24.13% and 23.39% were achieved under reverse and forward scans, respectively (Figure S6a). The integrated photocurrent density derived from the incident photon‐to‐current efficiency (IPCE) spectrum (Figure S6b) agrees well with that obtained from the *J*–*V* measurements, with a minimal mismatch of 2.9%. Figure 4a–c summarizes the statistical photovoltaic parameters of PSCs with and without MBA passivation. The control devices showed a large difference in *V*_OC_ between forward and reverse scans, consistent with the hysteresis behavior observed in the representative *J*–*V* curves. In contrast, MBA‐passivated devices exhibited nearly identical *V*_OC_ values in both scan directions, with the average *V*_OC_ (reverse scan) increasing from 1.08 to 1.12 V. Due primarily to this *V*_OC_ improvement, along with a slight enhancement in fill factor (FF), the average PCE (considering both forward and reverse scan directions) increased from 19.3% to 22.2%, corresponding to an average Δ*PCE*_norm_of 0.15, a value comparable to or exceeding that of previously reported passivation molecules (see Data T0). Furthermore, the *J*–*V* hysteresis index ($hysteresis\;index=\frac{PCE_{\mathrm{reverse}}-PCE_{\mathrm{forward}}}{PCE_{\mathrm{reverse}}}$) was significantly reduced, from 0.160 in control devices to 0.036 after MBA treatment. We further performed maximum power point tracking (MPPT) measurements on two representative devices exhibiting average efficiencies (19.2% for the reference and 22.4% for the passivated device). As shown in Figure S7, during 350 s of continuous tracking, the passivated device exhibits constant efficiency while the reference one decreases remarkably. This behavior is consistent with the reduced *J*–*V* hysteresis, as stabilized MPPT and suppressed hysteresis share a common origin in diminished interfacial trapping and ionic migration. These results indicate that MBA treatment not only passivates surface defects but also enhances interfacial carrier transport.

To further elucidate the role of MBA at the perovskite interface, perovskite films and devices with and without MBA treatment were systematically investigated. The interaction between MBA molecules and surface Pb^2+^ sites was examined by X‐ray photoelectron spectroscopy (XPS). As shown in Figure S8, the Pb 4*f* core‐level peaks shift toward higher binding energies (∼0.20 eV) after passivation, indicating strong coordination between the carboxylate groups of MBA and under‐coordinated Pb^2+^ sites. This interaction modifies the local electronic environment of Pb, directly confirming effective chemical passivation of surface defects [31]. Steady‐state photoluminescence (PL) and time‐resolved PL (TRPL) measurements were conducted on perovskite films deposited on glass substrates to isolate surface effects. As shown in Figure 4d,e, MBA‐passivated films exhibit stronger PL intensity and prolonged carrier lifetimes, confirming suppressed nonradiative recombination and reduced trap density. These findings are further supported by transient photovoltage (TPV) measurements (Figure S9a), where enhanced photovoltage amplitude and extended decay lifetimes are observed after passivation, indicating suppressed interfacial recombination and improved quasi‐Fermi level splitting [32], consistent with the observed *V*_OC_ enhancement.

The surface energetics of the perovskite films were characterized by UV–vis absorption spectroscopy and photoelectron yield spectroscopy (PYS) (Figure S10a–c). While the optical bandgap remains unchanged, the perovskite conduction band shifts downward from 5.39 to 5.46 eV after MBA treatment (Figure S10c), which might be attributed to the molecular dipole moment of MBA [33, 34]. The electronic properties of the PCBM electron transport layer were also determined by UV–vis and PYS measurements (Figure S9d–f). Based on these results, the energy‐level alignment diagram was constructed (Figure 4f). Consequently, the energy offset between the perovskite and PCBM layers is reduced from 0.07 eV to nearly zero, minimizing interfacial energy loss and accounting for the enhanced *V*_OC_. Moreover, this favorable alignment promotes faster electron extraction and suppresses interfacial charge accumulation, thereby reducing *J*–*V* hysteresis [32, 35, 36]. Consistently, transient photocurrent (TPC) measurements (Figure S9b) show faster current decay after passivation, confirming accelerated charge extraction [32].

To further support the experimental observations, density functional theory (DFT) calculations were performed to examine the interaction between representative passivation molecules and the perovskite surface (Figure S11). Among the three molecules considered, MBA exhibits the strongest interaction with the FAI‐terminated FAPbI_3_ (001) surface, with an adsorption energy of −1.462 eV and the largest charge transfer (0.146 e). Charge‐density difference plots reveal pronounced charge accumulation localized around the three oxygen atoms in the terminal functional group of MBA. In contrast, maleic acid monoamide and DL‐mandelic acid contain only two terminal oxygen atoms, resulting in weaker charge redistribution and smaller adsorption energies. These results suggest that the enhanced interaction strength of MBA arises from its molecular functionality and contributes to more effective defect passivation, consistent with previous reports correlating stronger adsorption with improved interfacial passivation [37].

In summary, combined experimental and theoretical results demonstrate that the proposed AI‐driven pipeline successfully identifies effective passivation candidates, such as MBA, capable of reducing surface defects in perovskite films, improving interfacial charge transfer, and enhancing overall device efficiency. These findings validate the reliability of the generative AI‐assisted molecular design strategy and highlight its strong potential for guiding future PSC optimization.

## 3 Conclusions

This work establishes an AI‐driven framework that accelerates the discovery of molecular passivators for PSCs. A discriminative model based on SMILES‐X successfully distinguished effective from ineffective molecules with high accuracy (F1 = 0.80, ROC‐AUC = 0.88), while a GPT‐2–based generative model iteratively produced over 100 000 chemically valid and largely effective candidates. Integrating these two models, for the first time, enabled exploration of previously inaccessible regions of chemical space. Multi‐criteria filtering and clustering reduced the candidates to ∼8000 high‐quality molecules while preserving chemical diversity. Among the three experimentally tested molecules, 4‐maleimidobutyric acid (MBA) delivered the most significant improvement, increasing the *V*_OC_ from 1.08 to 1.12 V and boosting the average power conversion efficiency from 19.3% to 22.2%, with an impressive Δ*PCE*_norm_of 0.15. These results validate the reliability of the proposed AI‐assisted molecular design strategy for PSCs. By reducing reliance on trial‐and‐error chemistry and enabling exploration of uncharted chemical space, this framework exemplifies how generative AI can complement traditional design methodologies and shorten the pathway from molecular concept to functional optoelectronic materials.

While the present framework demonstrates effective enrichment of candidate passivation molecules, several limitations should be noted. The discriminative model is trained on literature‐derived Δ*PCE*_norm_ values and therefore primarily captures empirical structure–performance correlations reflected in reported device outcomes. As such, it may combine transferable chemical motifs with interpolation within chemistries represented in the training data, rather than explicitly learning physics‐based descriptors. Incorporating systematic theoretical quantities as additional training labels would further enhance mechanistic interpretability and generalizability in future developments. In addition, because Data T0 was constructed from published studies, it may reflect inherent publication bias toward improved passivation strategies, while fully negative or null results remain underrepresented. Although the dataset spans a range of performance outcomes and includes cases treated as class 0, incorporating systematically collected negative samples would sharpen class boundaries and reduce potential label noise. Finally, the present AI workflow is efficiency‐oriented, as it is trained exclusively on efficiency‐based labels. The MPPT tracking characterizes operational efficiency rather than ISOS‐compliant lifetime evaluation. Future extensions integrating standardized stability metrics as training objectives would enable multi‐objective optimization of both efficiency and long‐term durability.

## Author Contributions

A.T.N.F., Z.G., and G.L. conceived the study. ATNF designed the computational methods, prepared the code, and performed the data analysis. G.L. assisted with data analysis and interpretation. Z.G. and R.G. conducted experimental measurements and T.M. associated data analysis. J.M. performed DFT calculations and associated data analysis. K.S. and R.F.P. contributed to data collection and preprocessing. A.T.N.F. and Z.G. drafted the manuscript, and all authors reviewed and approved the final version.

## Conflicts of Interest

The authors declare no conflicts of interest.

## Supporting information




**Supporting File**: advs75128‐sup‐0001‐SuppMat.pdf.

## Data Availability

The data that support the findings of this study are available in the supplementary material of this article. All code used in this study has been deposited at: https://github.com/adroitfajar/pvmol‐gen.
