# Supplementary material for: Generative AI‐Driven Accelerated Discovery of Passivation Molecules for Perovskite Solar Cells
Source: Adv Sci (Weinh). 2026 Apr 2;13(36):e23042. doi: 10.1002/advs.202523042 (PMC13317563; doi:10.1002/advs.202523042)
Supplement: Supplementary file 1 — Supporting File: advs75128‐sup‐0001‐SuppMat.pdf. [file ADVS-13-e23042-s001.pdf]

SUPPORTING INFORMATION

Generative AI-driven Accelerated Discovery of Passivation Molecules for Perovskite Solar Cells

Adroit T.N. Fajar[1, 2]*, Guillaume Lambard[3], Jessie Manopo[1, 2], Ruili Guo[1], Kevin Septioga[4], Rizfi F. Pari[4], Toshinori Matsushima[1, 2, 4, 5], Zhanglin Guo[1, 2, 5]*

[1]*International Institute for Carbon-Neutral Energy Research (WPI-I²CNER), Kyushu University, 744 Motooka, Fukuoka 819-0395, Japan.*
[2]*Center for Energy Systems Design (CESD), International Institute for Carbon-Neutral Energy Research (WPI-I²CNER), Kyushu University, 744 Motooka, Fukuoka 819-0395, Japan.*
[3]*Data-driven Materials Design Group, Center for Basic Research on Materials, National Institute for Materials Science, Namiki 1-1, Tsukuba 305-0044, Japan.*
[4]*Department of Applied Chemistry, Graduate School of Engineering, Kyushu University, 744 Motooka, Fukuoka 819-0395, Japan.*
[5]*Department of Automotive Science, Graduates School of Integrated Frontier Sciences, Kyushu University, 744 Motooka, Nishi, Fukuoka 819-0395, Japan*

*Corresponding Authors:
adroit@i2cner.kyushu-u.ac.jp (AF); guo.zhanglin.903@m.kyushu-u.ac.jp (ZG)

METHODS

1. Dataset preparation

The initial dataset (Data T0) was constructed by systematically mining passivation molecules summarized in several widely cited articles covering molecular passivation strategies for PSCs published between 2016–2025,[1–5] which were used as entry points to trace the corresponding primary studies. Rigorous preprocessing was performed to the collected data to ensure compatibility with the models used in this study. The preprocessing pipeline included: (i) filtering out ionic compounds, (ii) adding recent publications up to March 2025, (iii) converting all passivation molecules to canonical Molecular Input Line Entry System (SMILES) representations, and (iv) removing duplicate entries. Polymer passivators were excluded because the present workflow focuses on discrete molecules representable by SMILES. This process yielded 314 unique molecules, each labeled with both initial and final power conversion efficiency (PCE) values, from which the relative (normalized) change in PCE (ΔPCE_{norm}) was calculated as described in Equation S1 (PCE_f and PCE_i represent the final and initial PCE values, respectively). When a molecule was reported in multiple publications, the instance corresponding to the highest reported ΔPCE_{norm} was retained.

$$\Delta PCE_{norm} = \frac{PCE_f - PCE_i}{PCE_i} \qquad (S1)$$

Raw data sources, including DOIs for each original publication, are provided in the dataset file (`dataset.xlsx, containing 12 sheets`) available as a Supporting Information file. An augmented dataset (Data T-aug) was prepared by retrieving molecules from the PubChem Compound database with >80% structural similarity to molecules showing ΔPCE_{norm} values above 0.16 in Data T0. The cheminformatics toolkit RDKit was employed to verify SMILES correctness before model training.[6]

2. Discriminative models

The LM-based molecular characterization tool SMILES-X, introduced by Lambard et al., [7] was utilized to build a binary classification model based on Data T0. SMILES-X does not require molecular descriptors and directly uses the SMILES strings as input features, mapping them to binary classification labels. The model was then used to predict the binary class (0 or 1) of previously unseen molecules. The optimal probability threshold of 0.47, determined from validation on Data T0 to maximize the F1 score, was consistently applied for all downstream binary classifications, including labeling of the augmented dataset (Data T-aug) and evaluation of generated molecules (Data G1–G3). As a comparison, a classification model based on a random forest (RF) classifier with fingerprint-based features was prepared. Further details of the SMILES-X and RF training and evaluation procedures are provided below:

2.1. SMILES-X classifier

The SMILES-X architecture was configured with three primary hyperparameters: the size of the embedding layer and the number of units in both the LSTM and dense layers, with possible values of [8, 16, 32, 64, 128, 256, 512, 1024]. These hyperparameters were optimized using zero-cost geometry optimization to automatically identify the optimal architecture. For further optimization, the batch size and learning rate (in powers of 10) were determined via Bayesian optimization, with search regions set to [8, 16, 32, 64] for batch size and [2.0, 2.5, 3.0, 3.1, 3.2, ..., 4.0] for learning rate. The models were trained for up to 100 epochs, with generalization capabilities evaluated using *k*-fold cross-validation (`k = 5`). Each fold in the k-fold cross-validation was repeated three times using different random seeds (3 runs per fold). The reported performance metrics are averaged over these runs to ensure a reliable estimation of model generalization and to provide information on standard deviation. To improve the model's robustness, two additional features were appended to each SMILES representation, specifically the number of hydrogen bond acceptors (`hba_num`) and the number of oxygen atoms (`o_num`), calculated using RDKit. The training was performed on a single NVIDIA RTX A5000 GPU, and the overall workflow, including hyperparameter optimization, model training, and evaluation, took approximately 3 hours to complete.

2.2. RF classifier

For comparison, a random forest (RF)-based classification model was developed using molecular fingerprints and auxiliary features. Canonical SMILES strings were converted to 2048-bit Morgan fingerprints (`radius = 2`) using RDKit. These fingerprint vectors were concatenated with the `hba_num` and the `o_num` to form the final feature set. The data were split into training and testing subsets using an 80:20 ratio. Hyperparameter tuning was performed via grid search with *k*-fold cross-validation (`k = 5`). The search space included:

number of estimators (`n_estimators` = [100, 200, 300]), tree depth (`max_depth` = [5, 10, 15]), and node constraints (`min_samples_split` = [2, 5, 10]; `min_samples_leaf` = [1, 2, 4]). The model showing the best cross-validated performance was selected. Additional evaluations were conducted on the test set and using full 5-fold cross-validation. The full training and evaluation process took approximately 2 hours using an Intel Xeon Silver CPU (48 cores, parallelized with `n_jobs=-1`).

2.3. Evaluation metrics

Model performance was assessed using standard binary classification metrics, including precision, recall, F1 score, and the area under the receiver operating characteristic curve (ROC-AUC). These were calculated as:

$$Precision = \frac{TP}{TP + FP} \qquad (S2)$$

$$Recall = \frac{TP}{TP + FN} \qquad (S3)$$

$$F1\ Score = 2 \cdot \frac{Precision \cdot Recall}{Precision + Recall} \qquad (S4)$$

$$ROC - AUC = \int_{0}^{1} TPR(FPR)d(FPR) \qquad (S5)$$

Here, TP, FP, FN refer to true positives, false positives, and false negatives, respectively; TPR is the true positive rate; and FPR is the false positive rate. All metrics were computed on both the held-out test set and using 5-fold cross-validation. Confusion matrices and precision–recall curves were generated to visualize classification performance.

3. Generative models

A dataset for training the generative models was constructed by collecting class 1 molecules from Data T0 and Data T-aug, resulting in 11,086 unique entries, designated as Data T1. LM-based generative models were then developed by fine-tuning GPT-2 (≈124 million parameters)[8] and LLaMA-2 (≈7 billion parameters)[9] on the SMILES strings in Data T1. These fine-tuned models were tasked with generating correct, unique, and novel (CUN) SMILES strings, targeting a total of 100,000 molecules, with a maximum of 10 million inference attempts permitted to achieve the desired quality. During generation, RDKit was employed to validate each molecule and filter out invalid SMILES strings, charged species, radicals, metal-containing compounds, and duplicate entries. The resulting dataset of molecules generated by the fine-tuned GPT-2 model was designated as Data G1. Further details of the fine-tuning and inference procedures for GPT-2 and LLaMA-2 are provided below.

3.1. GPT-2 fine-tuning and inference

Prior to training, each SMILES was augmented into multiple non-canonical variants to enhance representation diversity. The SMILES were tokenized using a modified GPT-2 tokenizer, which included a custom padding token. The tokenized sequences were split into an 80:20 training and validation set. Training was performed for up to 100 epochs using the HuggingFace `Trainer` API with an early stopping criterion (`patience = 5 epochs`). The training was executed using mixed canonical/non-canonical SMILES, with a batch size of 2 and weight decay of 0.01. The best model checkpoint was selected based on evaluation loss. The training objective is to minimize the standard cross-entropy loss between the predicted and target SMILES tokens:

$$\mathcal{L}_{CE} = -\sum_{t=1}^{T} \log_{p\theta}(x_t | x_1, x_2, \cdots, x_{t-1}) \qquad (S6)$$

The final model and tokenizer were saved and used in downstream inference. For molecule generation, the fine-tuned GPT-2 model was used to perform stochastic sampling via nucleus sampling (`temperature = 0.9, max length = 100`). Each generated string was decoded and validated using RDKit to ensure chemical correctness. Invalid, duplicate, charged, radical-containing, or metal-containing SMILES were filtered out. Generation continued until the target number of correct-unique-novel (CUN) SMILES strings was obtained, with a maximum cap of 100× the target number of inference attempts to guarantee diversity and uniqueness.

- Top-k sampling restricts choices to the *k* highest-probability tokens:

$$V_k = \arg\ top\_k\ (p(x) | x \in V) \qquad (S7)$$

- Nucleus (top-p) sampling selects from the smallest token set $V_p \subset V$ such that:

$$\sum_{x \in V_p} p(x) \geq p \qquad (S8)$$

Both training and inference were handled using PyTorch and performed on four NVIDIA A5000 GPUs. Training typically took 24–48 hours to complete, and inference took about a week to generate 100,000 CUN molecules.

3.2. LLaMA-2 fine-tuning and inference

For comparison, another generative model was developed using LLaMA-2 (7 billion parameters), fine-tuned on the same augmented SMILES dataset (Data T1). Training was conducted using the HuggingFace `Trainer` API in full precision (FP32). The tokenizer was modified to support padding and long sequences (up to 512 tokens). The model was trained for up to 100 epochs using a small batch size (1) with gradient accumulation steps = 4 to emulate a larger effective batch. Early stopping (`patience = 3 epochs`) was applied. Learning rate was set to 1e-5 with weight decay of 0.01. The fine-tuned model and tokenizer were saved for inference. Generation with the fine-tuned LLaMA-2 model followed a similar pipeline to GPT-2, using top-k (k = 50) and top-p (p = 0.95) sampling. Output sequences were validated using RDKit in the same manner as the GPT-2 model.

Both training and inference were handled using PyTorch and performed on four NVIDIA A100 GPUs on the Genkai Supercomputer (https://www.cc.kyushu-u.ac.jp/scp/). Training typically took 72–96 hours to complete, and inference took about a week to generate 1,000 CUN molecules.

3.3. Post-processing after generation

After generation, a post-processing pipeline was applied to ensure novelty and consistency of the generated SMILES. First, each molecule was converted to its canonical SMILES form using RDKit, and duplicates were removed. The resulting set was then compared against the training dataset to filter out previously seen molecules. SMILES entries found to be duplicates of training molecules were excluded, and only novel molecules were retained for downstream analysis. The final output was saved as a CSV file, containing the filtered, unique, and novel SMILES. Additionally, a subset of molecules was visualized to confirm structural diversity and validity.

3.4. Iterative training and inference

To generate diverse and high-quality molecules biased toward class 1, an iterative training and inference process was conducted following our previous methodology.[10] The SMILES-X classification model was first used to predict the binary class of molecules in Data G1, and all molecules classified as class 1 were added to Data 1, expanding the training set to 34,538 unique SMILES entries, designated as Data T2. Subsequently, the GPT-2 model was re-trained on Data T2, and the updated model was used to generate a new set of molecules, designated as Data G2. This iterative process was repeated for three cycles, ultimately producing over 100,000 generated molecules with a bias toward class 1 candidates.

4. Filtering criteria

A two-stage workflow was employed to filter the generated molecules based on seven physicochemical property windows. First, each SMILES string was processed to calculate the synthetic accessibility (SA) score, presence of PAINS substructure alerts, hydrogen-bond donor (HBD) count, hydrogen-bond acceptor (HBA) count, and topological polar surface area (TPSA). In parallel, a single 3D conformer was generated for each molecule using the ETKDG algorithm, followed by a single-point xTB calculation to estimate the HOMO–LUMO gap. Concurrently, Gasteiger partial charges were computed on the same conformer and used to approximate the molecular dipole moment via the vector sum of atomic charges and coordinates. Subsequently, hard filters were applied with the following thresholds: SA $\leq$ 6; absence of PAINS; $2 \leq$ HBA ≤ 5; $0 \leq$ HBD ≤ 2; 50 $Å^2 \leq$ TPSA $\leq$ 120 $Å^2$; HOMO–LUMO gap between 1.5–5.0 eV; and dipole moment between 1.5–4.0 D. This resulted in a reduced candidate library. Finally, agglomerative clustering was performed using Morgan fingerprints (radius = 2, 1024 bits) with Tanimoto distance as the similarity metric. The number of clusters was set to 10 to balance structural diversity and feasibility of experimental validation. One representative molecule was randomly selected from each cluster to ensure coverage of distinct regions of the candidate space.

5. Experimental studies

Inverted perovskite solar cells (PSCs) were fabricated to experimentally verify the effectiveness of the AI-generated passivation molecules. Indium tin oxide (ITO) glass substrate (2.5 × 2.5 cm^2) was ultrasonically cleaned with water, acetone, and 2-proponal in sequence. Before use, the ITO substrates were treated with UV–ozone for 15 min. A 10 mg mL^{-1} NiO_x solution was prepared by dispersing NiO_x nanoparticles (Advanced Election Technology Co., China) in a mixed solvent of H_2O and 2-propanol (v/v = 3:1). The solution was spin-coated onto ITO, followed by drying at 120 °C for 20 min in air. Subsequently, the substrates were transferred to an N_2-filled glovebox. A co-self-assembled monolayer (co-SAM) was formed by spin-coating a mixed solution containing 0.85 mg 2PACz and 1.70 mg Me-4PACz (Tokyo Chemical Industry, Japan) dissolved in 5 mL ethanol onto the NiO_x layer. The coated film was then dried at 100 °C for 10 min and dynamically rinsed with ethanol to remove unbound molecules. It should be noted that, for simplicity, the term "SAM" is used here, although materials such as 2PACz and Me-4PACz may not form a true self-assembled monolayer but rather a covalently bonded layer to a metal oxide substrate, and direct evidence for ordered monolayer formation is also lacking. 1.6 M perovskite precursor solution with the composition $Cs_{0.05}FA_{0.95}PbI_3$ was prepared by dissolving CsI, FAI, and PbI_2 in a DMF:DMSO mixed solvent (v/v = 4:1). To improve device performance, 5 mol% of $PbCl_2$ and 15 mol% MACl were added. All the above chemicals are purchased from TCI, Japan. 90 μL of the perovskite solution was spin-coated onto the co-SAM substrates using a two-step program: 2000 rpm for 10 s and 4000 rpm for 45 s. During the second step, 180 μL chlorobenzene was dripped at 5 s before the end of spin-coating. The films were then annealed at 100 °C for 30 min.

The passivation molecule solution (1 mg mL^{-1} in 2-propanol) was dynamically spin-coated on perovskite surface at 5000 rpm for 30 s, followed by annealing at 100 °C for 5 min. A 20 mg mL^{-1} of PCBM solution (Sigma-Aldrich) in chlorobenzene was spin-coated at 4000 rpm for 30 s and annealed at 70 °C for 10 min. Subsequently, a 1.5 mg ml^{-1} of BCP solution in methanol was spin-coated at 6000 rpm for 30 s and annealed at 70 °C for 5 min. Finally, a 100 nm thick Ag electrode was thermally evaporated through a shadow mask (active area = 0.12 cm^2) to complete the inverted PSC fabrication. For film characterizations, perovskite layers were deposited on either ITO or glass substrates, followed by coating of the passivation layer using the same conditions as those employed in device fabrication.

6. First principles calculations

First-principles calculations were performed using Quantum ESPRESSO.[11] The generalized gradient approximation with the Perdew–Burke–Ernzerhof (PBE) exchange–correlation functional was employed for all calculations.[12] A vacuum layer of approximately 35 Å was applied along the surface normal to avoid spurious interactions between periodic images. The surface model was constructed using a 2 × 2 × 1 supercell of the $FAPbI_3$ (001) surface. A 3 × 3 × 1 Monkhorst–Pack k-point mesh and a plane-wave energy cutoff of 50 Ry were used. The FAI-terminated surface, which has been reported to exhibit the lowest surface energy, was considered.[13] Charge-density difference plots were calculated as

$$\Delta\rho = \rho_{surface+molecule} - \rho_{surface} - \rho_{molecule} \qquad (S9)$$

and visualized using an isosurface value of 0.001 e $Å^{-3}$. Adsorption energies (E_{ads}) were computed to quantify molecule–surface interaction strength.

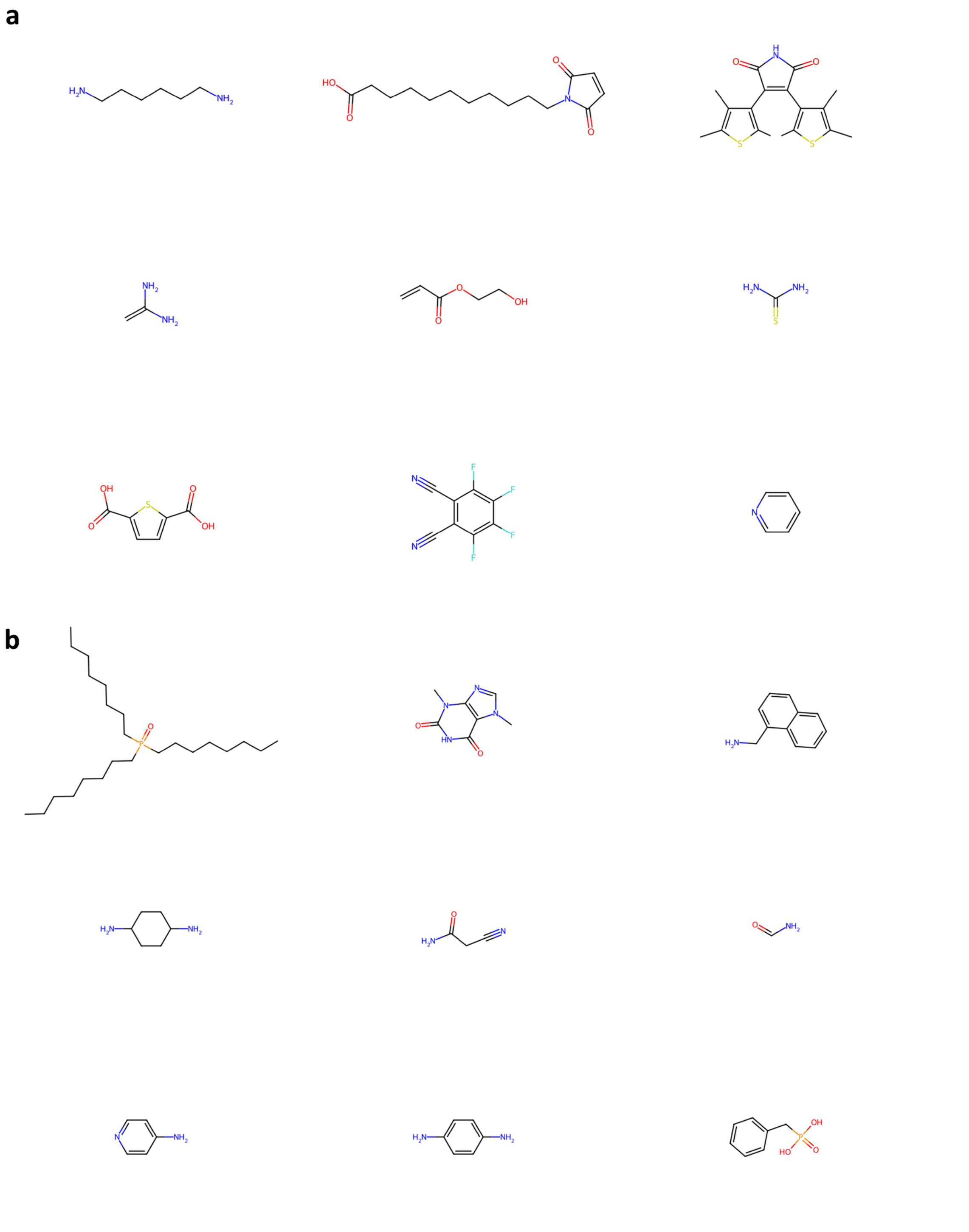

Figure S1. Examples of molecular structures from (a) class 1 and (b) class 0, where molecular scaffolds or substructures are not readily distinguishable by visual inspection.

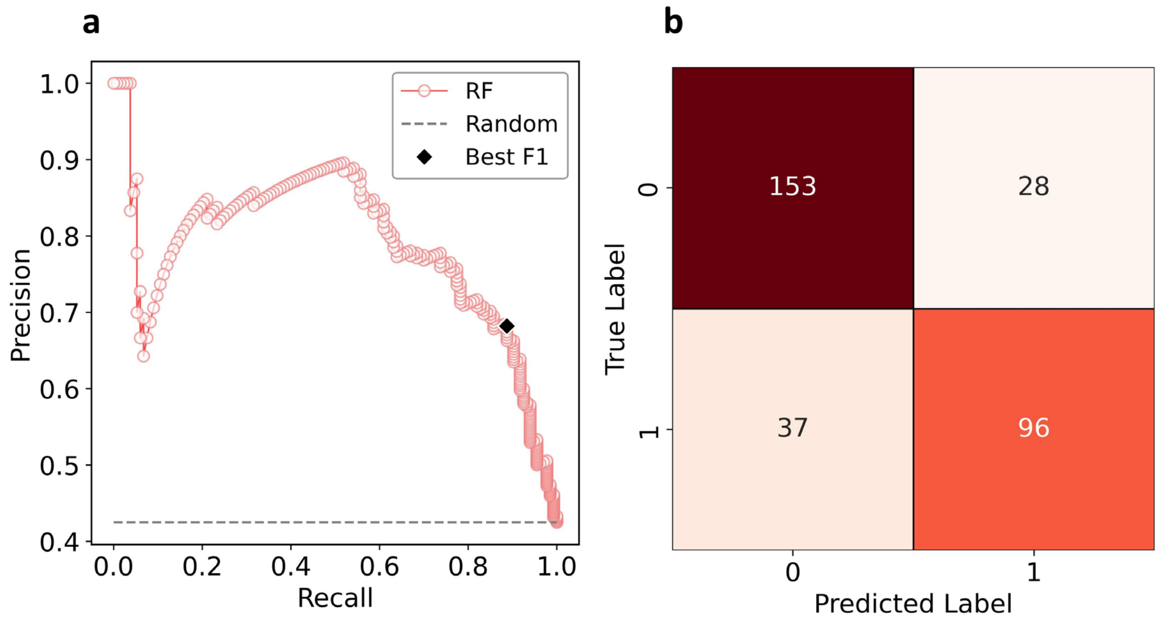

Figure S2. (a) Precision–recall (PR) curve and (b) confusion matrix of the binary classification model prepared using the random forest (RF) algorithm trained on Data T0.

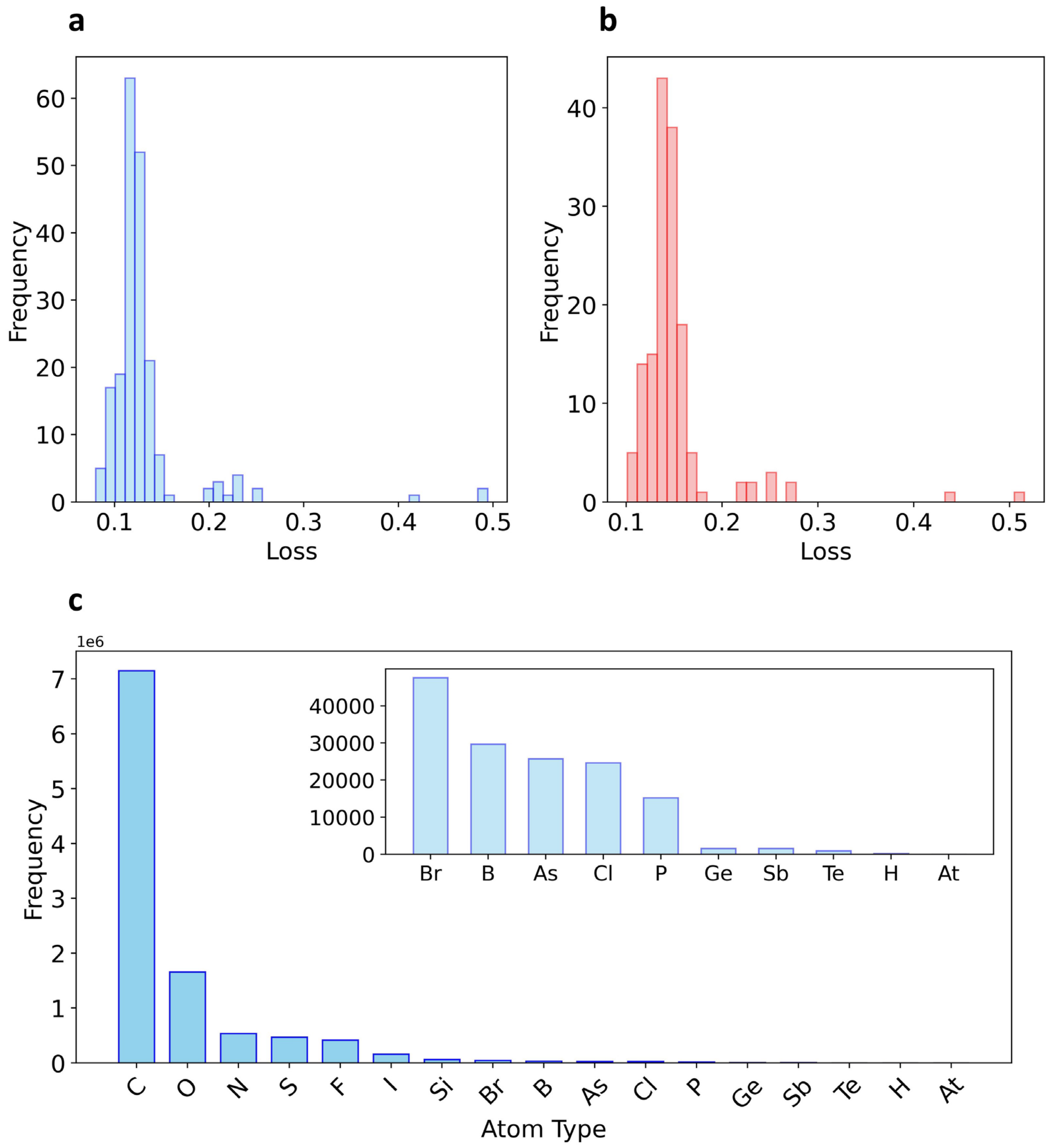

Figure S3. Test loss distributions upon fine-tuning (a) GPT-2 and (b) LLaMA-2 models with Data T1. (c) Distribution of atom types in the cumulative set of generated molecules (Data G-all).

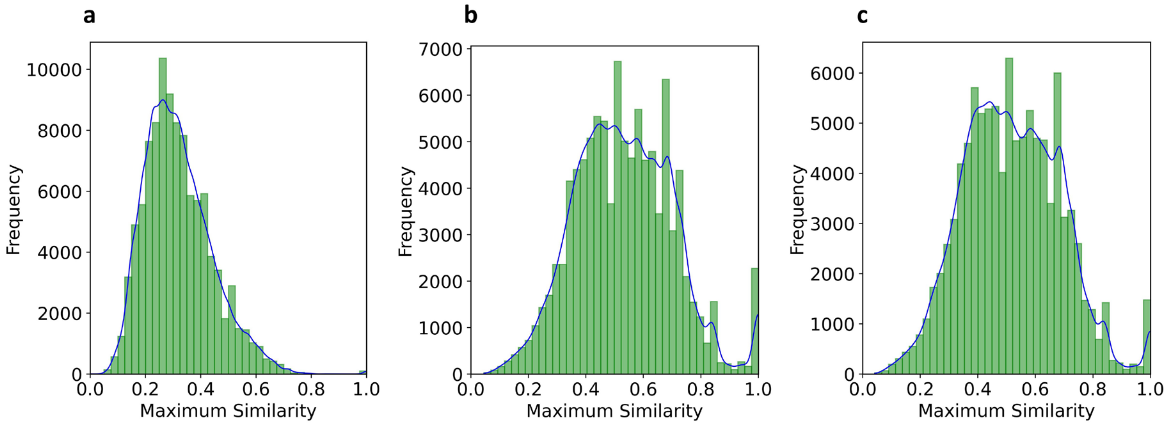

Figure S4. Distribution of maximum Tanimoto similarity (Morgan fingerprints, radius = 2, 2048 bits) between the cumulative generated library (Data G-all) and (a) the seed dataset (Data T0), (b) the augmented dataset (Data T-aug), and (c) the GPT-2 fine-tuning dataset (Data T1). The similarity distributions are centered at moderate values, indicating that the generated molecules explore chemically related but non-identical regions of chemical space. For the comparison with the training dataset (T1), the distribution is centered at moderate similarity values (mean = 0.520, median = 0.513), with over 85% of generated molecules below 0.7 similarity and fewer than 2% showing exact matches (similarity = 1.0), indicating substantial chemical diversification beyond the training distribution.

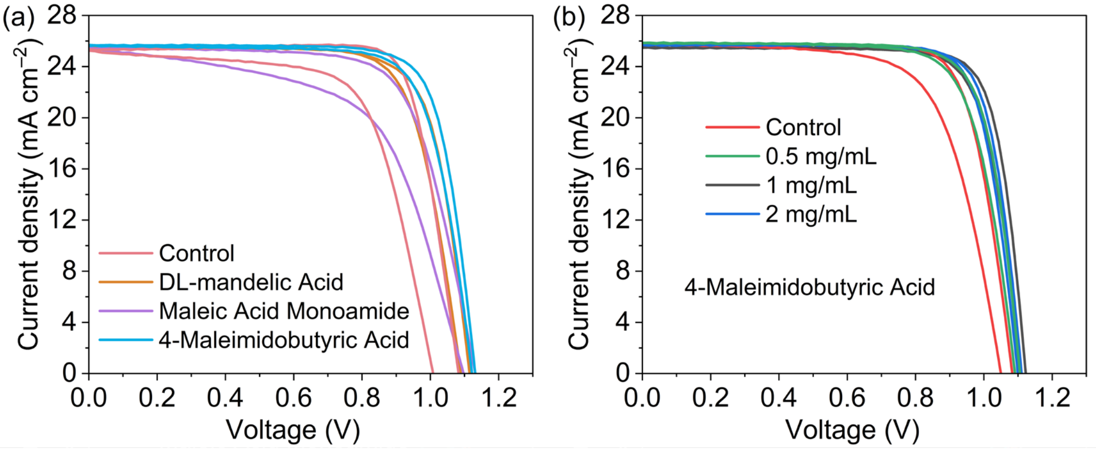

Figure S5. (a) *J-V* curves of inverted perovskite solar cells with and without passivation; (b) *J-V* curves of 4-Maleimidobutyric Acid passivated inverted perovskite solar cells with different concentrations.

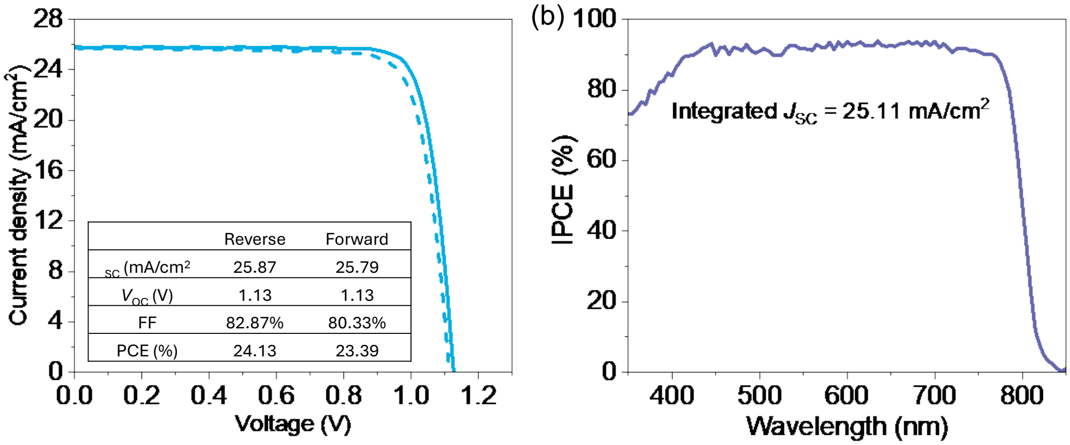

Figure S6. (a) *J-V* curves and (b) IPCE of the champion device with 4-Maleimidobutyric Acid passivation.

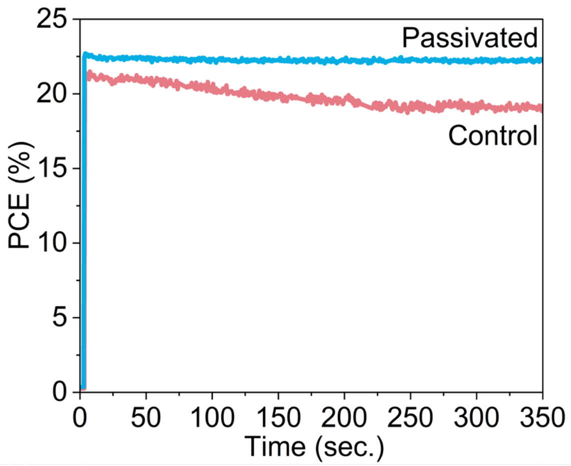

Figure S7. Maximum power point tracking (MPPT) of representative control and 4-Maleimidobutyric Acid passivated solar cells.

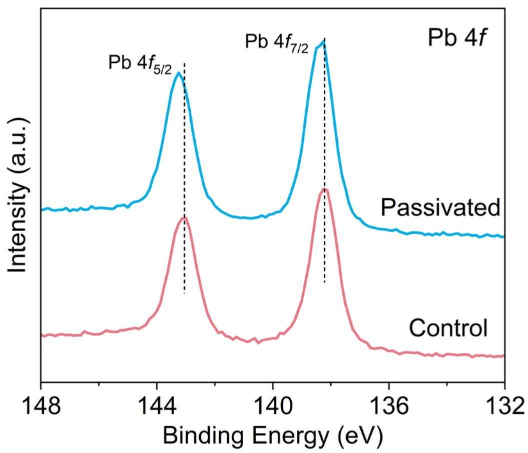

Figure S8. XPS Pb 4*f* spectra of control and 4-Maleimidobutyric Acid passivated perovskite films.

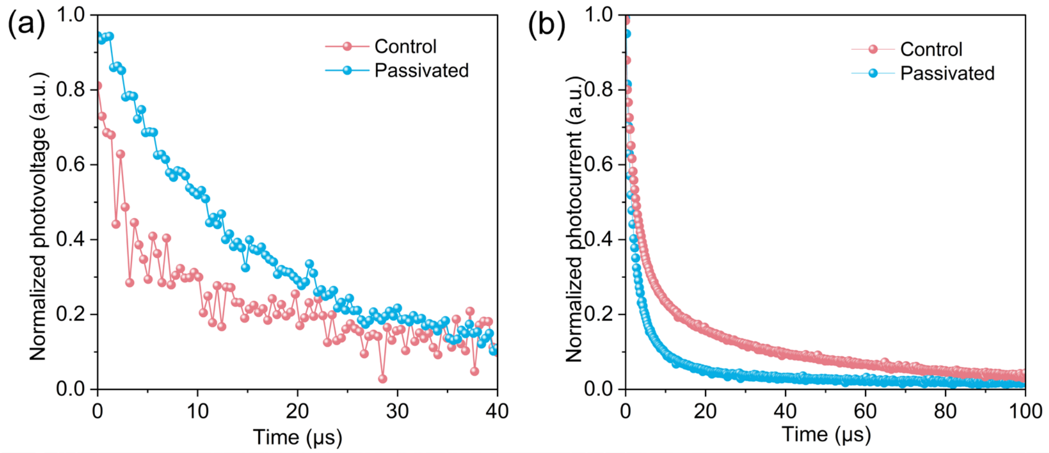

Figure S9. (a) Transient photovoltage (TPV) and (b) transient photocurrent (TPC) spectra of control and 4-Maleimidobutyric Acid passivated solar cells.

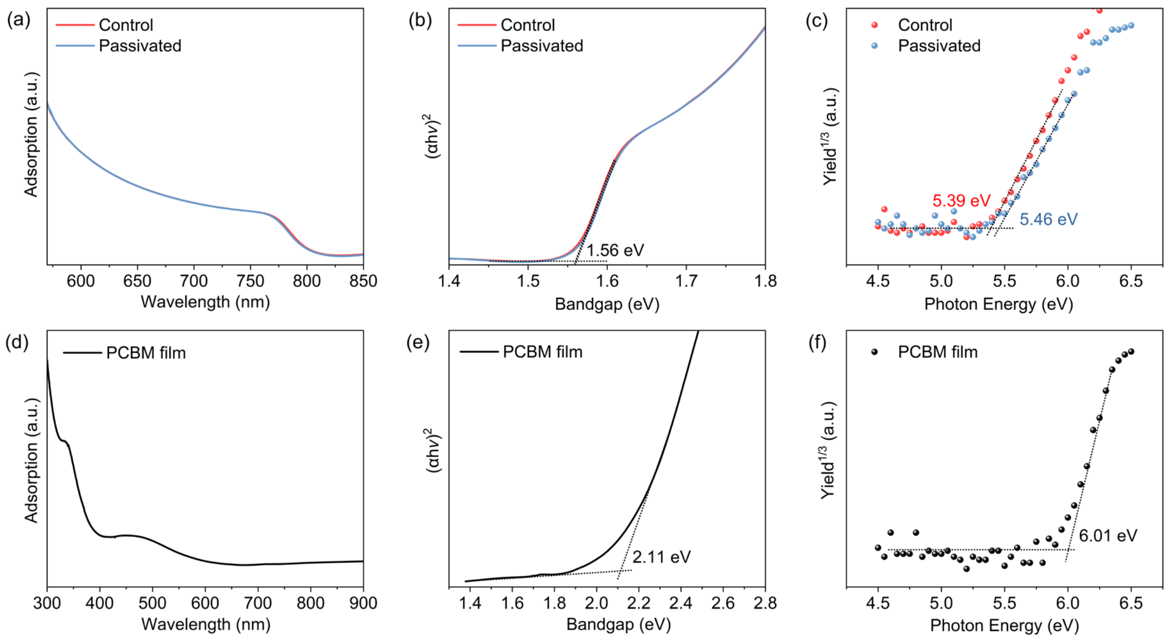

Figure S10. Characterizations of perovskite films on ITO substrate with and without 4-Maleimidobutyric Acid passivation (1 mg/mL): (a) UV-Vis spectra, (b) Tau plots, and (c) PYS spectra; characterizations of PCBM film deposited on ITO substrate (20 mg/mL): (d) UV-Vis spectrum, (e) Tau plot, and (f) PYS spectrum.

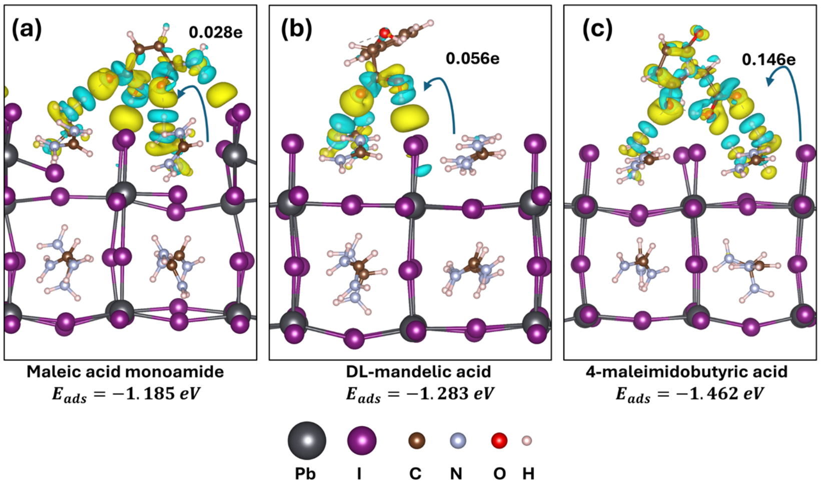

Figure S11. Charge-density difference plots for adsorption of (a) maleic acid monoamide, (b) DL-mandelic acid, and (c) 4-maleimidobutyric acid (MBA) on the FAI-terminated $FAPbI_3$ (001) surface. Yellow and turquoise regions indicate charge accumulation and depletion, respectively. The corresponding adsorption energies E_{ads} are shown for each molecule.

Table S1. Concise sensitivity analysis for the classifier threshold.

Threshold	Class-1 fraction (%)	Class-1 candidate pool
0.45	86.12	91,532
0.47	82.55	87,750
0.50	80.55	85,620
0.55	78.04	82,942

Table S2. Representative molecules randomly selected from each cluster.

Index	SMILES	SA	PAINS	HBA	HBD	TPSA	Gap	Dipole
S1	O=C(O)c1ccc(/C=C\CO)s1	2.64	False	3	2	57.53	2.60	2.05
S2	O=C(O)C(F)(F)C(=O)c1cccs1	2.73	False	3	1	54.37	2.84	2.39
S3	NC(=O)/C=C/CC(=O)O	3.80	False	2	2	80.39	3.34	2.72
S4	O=S(=O)(c1ccccc1)n1cc(C2=CCNCC2)c2cc(F)ccc21	2.52	False	4	1	51.10	2.21	3.03
S5	O=C(O)c1ccccc1Nc1ccccn1	1.69	False	3	2	62.22	2.64	2.45
S6	O=C(Cc1ccccc1O)c1c[nH]c2ccccc12	1.93	False	2	2	53.09	2.47	3.73
S7	O=C(Nc1ccccc1C(=O)Nc1ccccc1)c1ccccc1	1.40	False	2	2	58.20	2.98	2.29
S8	O=C(O)c1cc(Br)c(C(=O)O)s1	2.51	False	3	2	74.60	2.37	1.74
S9	O=C(O)C(O)c1ccccc1	2.06	False	2	2	57.53	4.04	1.93
S10	CCC(C(=O)O)N1C(=O)C=CC1=O	3.16	False	3	1	74.68	2.27	2.53

Table S3. Molecules selected for evaluation in PSC passivation.

Index	CAS No.	Name	Structure	Similarity	Referred
C3	557-24-4	Maleic Acid Monoamide	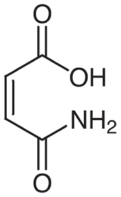	0.85	S3
C9	90-64-2	DL-Mandelic Acid	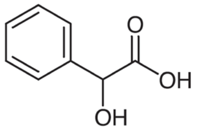	1.00	S9
C10	57078-98-5	4-Maleimidobutyric Acid	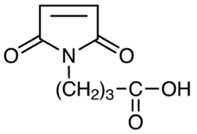	1.00	S10

REFERENCES

[1] Liu, W., Lu, Y., Wei, D., Huo, X., Huang, X., Li, Y., ... & Song, D. (2022). Screening interface passivation materials intelligently through machine learning for highly efficient perovskite solar cells. *Journal of Materials Chemistry A*, 10(34), 17782-17789. https://doi.org/10.1039/D2TA04788H

[2] Zhi, C., Wang, S., Sun, S., Li, C., Li, Z., Wan, Z., ... & Liu, Z. (2023). Machine-learning-assisted screening of interface passivation materials for perovskite solar cells. *ACS Energy Letters*, 8(3), 1424-1433. https://doi.org/10.1021/acsenergylett.2c02818

[3] Zhang, X., Ding, B., Wang, Y., Liu, Y., Zhang, G., Zeng, L., ... & Chen, B. (2024). Machine learning for screening small molecules as passivation materials for enhanced perovskite solar cells. *Advanced Functional Materials*, 34(30), 2314529. https://doi.org/10.1002/adfm.202314529

[4] Chen, B., Rudd, P. N., Yang, S., Yuan, Y., & Huang, J. (2019). Imperfections and their passivation in halide perovskite solar cells. *Chemical Society Reviews*, 48(14), 3842-3867. https://doi.org/10.1039/C8CS00853A

[5] Gao, F., Zhao, Y., Zhang, X., & You, J. (2020). Recent progresses on defect passivation toward efficient perovskite solar cells. *Advanced Energy Materials*, 10(13), 1902650. https://doi.org/10.1002/aenm.201902650

[6] RDKit: Open-source cheminformatics. https://www.rdkit.org.

[7] Lambard, G., & Gracheva, E. (2020). SMILES-X: autonomous molecular compounds characterization for small datasets without descriptors. *Machine Learning: Science and Technology*, 1(2), 025004. https://doi.org/10.1088/2632-2153/ab57f3

[8] Radford, A., Wu, J., Child, R., Luan, D., Amodei, D., & Sutskever, I. (2019). Language models are unsupervised multitask learners. *OpenAI blog*, 1(8), 9.

[9] Touvron, H., Martin, L., Stone, K., Albert, P., Almahairi, A., Babaei, Y., ... & Scialom, T. (2023). Llama 2: Open foundation and fine-tuned chat models. *arXiv preprint arXiv:2307.09288*. https://doi.org/10.48550/arXiv.2307.09288

[10] Fajar, A. T., Lambard, G., Islam, M. A., Saha, B. B., Nurfajrin, Z. D., & Septioga, K. (2025). Generating eco-friendly ionic liquids with enhanced CO2 solubility using language models. *Artificial Intelligence Chemistry*, 3(1), 100089. https://doi.org/10.1016/j.aichem.2025.100089

[11] Giannozzi, P., Baroni, S., Bonini, N., Calandra, M., Car, R., Cavazzoni, C., ... & Wentzcovitch, R. M. (2009). QUANTUM ESPRESSO: a modular and open-source software

project for quantum simulations of materials. *Journal of physics: Condensed matter*, 21(39), 395502. https://doi.org/10.1088/0953-8984/21/39/395502

[12] Perdew, J. P., Burke, K., & Ernzerhof, M. (1996). Generalized gradient approximation made simple. *Physical review letters*, 77(18), 3865. https://doi.org/10.1103/PhysRevLett.77.3865

[13] Oner, S. M., Sezen, E., Yordanli, M. S., Karakoc, E., Deger, C., & Yavuz, I. (2022). Surface defect formation and passivation in formamidinium lead triiodide (FAPbI3) perovskite solar cell absorbers. *The Journal of Physical Chemistry Letters*, 13(1), 324-330. https://doi.org/10.1021/acs.jpclett.1c03645
